# Seasonality and trends in incidence of human ehrlichiosis in two Missouri ecoregions

**DOI:** 10.1017/S0950268818003448

**Published:** 2019-03-08

**Authors:** K. E. Andrews, K. K. Eversman, S. A. Foré, H. J. Kim

**Affiliations:** 1 Department of Biology, Truman State University, Kirksville, MO, USA; 2 Department of Mathematics, Truman State University, Kirksville, MO, USA; 3 Department of Statistics, Truman State University, Kirksville, MO, USA

**Keywords:** *Ehrlichia chaffeensis*, ehrlichiosis, incidence, Missouri, risk, season, ticks

## Abstract

Ehrlichiosis is a zoonotic illness caused by *Ehrlichia* pathogens transmitted by ticks. Case data from 1999 to 2015, provided by the Missouri Department of Health and Senior Services (DHSS), were used to compare the seasonality and the change in incidence over time of ehrlichiosis infection in two Missouri ecoregions, Eastern Temperate Forest (ETF) and Great Plains (GP). Although the number of cases has increased over time in both ecoregions, the rate of change was significantly faster in ETF region. There was no significant difference in seasonality of ehrlichiosis between ecoregions. In Missouri, the estimated ehrlichiosis season begins, on average, in mid-March, peaks in June, and concludes in mid-October. Our results show that the exposure and risk season for ehrlichiosis in Missouri is at least 7 months long.

## Introduction

Human ehrlichiosis is a zoonotic infection transmitted by ticks. The disease became nationally notifiable to the Center of Disease Control and Prevention (CDC) in 1999. Since the initial description of ehrlichiosis in 1986 [1], there has been an increase in the number of confirmed cases reported in the USA [2, 3]. Cases of ehrlichiosis have primarily been observed in south-central and south-eastern USA with the highest incidence occurring in Missouri, Arkansas, Delaware, Tennessee, Virginia and Oklahoma [3]. However, cases of ehrlichiosis have been reported as far south as Florida and as far north as Minnesota and Maine [3].

In 2008, the case definition was changed to create four sub-categories; infection by *Anaplasma phagocytophilum*, *Ehrlichia chaffeensis*, *E. ewingii* or undetermined (https://wwwn.cdc.gov/nndss/conditions/ehrlichiosis-and-anaplasmosis/). This change allowed for the differentiation between anaplasmosis and ehrlichiosis, as well as the categorisation of the closely related *E. chaffeensis* and *E. ewingii* pathogens that cause human ehrlichiosis. Both *E. chaffeensis*, the most common agent of infection, and *E. ewingii* are transmitted by *Amblyomma americanum*, the lone star tick [4].

Recent review of county records [5] shows that *A. americanum*, the lone star tick has a broader range than previously reported with established populations throughout the southeast into Texas, up into Minnesota and along the East Coast up in to New York. Ticks acquire the pathogen after taking a blood meal from an infected host. This tick is a non-specific and an aggressive feeder throughout all three life stages [6, 7]. In the USA, white-tailed deer (*Odocoileus virginianus)* are considered the most important reservoir host of *E. chaffeensis* [8] and *E. ewingii* [9] and can feed all three stages of the lone star tick. However, there is some evidence to suggest that dogs may serve a reservoir of *E. ewingii* [10]. Infected humans are incidental hosts and do not further transmit the pathogen to other organisms [11]. As *E. chaffeensis* does not exhibit transovarial transmission [12], only the nymph and adult life stages may transmit the pathogen. Adult ticks are infected with *E. chaffeensis* [13, 14] and *E. ewingii* [14] at higher rates. However, nymphs are more prevalent and less conspicuous than adults, and therefore may pose a greater risk of transmission to humans.

The primary objective of this study is to identify the season in which the majority of ehrlichiosis occurs in Missouri. As the seasonality of *E. chaffeensis* in the lone star tick has been associated with meteorological and environmental conditions such as moisture, temperature, habitat type and host density [15], we will explore differences in disease seasons between the Missouri Level I ecoregions: ETF, an area predominating the southern half of Missouri and characterised by deciduous trees, and the GP, an area predominating the northern half of the state characterised by agriculture and grasslands. This study will examine a small region of the lone star tick range; however, there may still be variation in the average season for ehrlichiosis due to differences in environmental conditions between the Missouri ecoregions that might influence exposures of humans to host seeking ticks. Environmental differences between the ecoregions may also affect change in disease incidence over time.

## Methods

The Missouri DHSS provided a dataset of confirmed cases of ehrlichiosis in Missouri from 1999 to 2015 by county. DHSS did not begin rigorous quality checks until 2005 and some of the early PCR tests did not differentiate *Ehrlichia* species. Although the causative agent of some of the cases was not established and might have been *E. wingii*, the number of reported cases of *E. wingii* cases from 2008 to 2012 was only 55 compared with 4613 cases of *E. chaffeensis* [3].

**Fig. 1.**
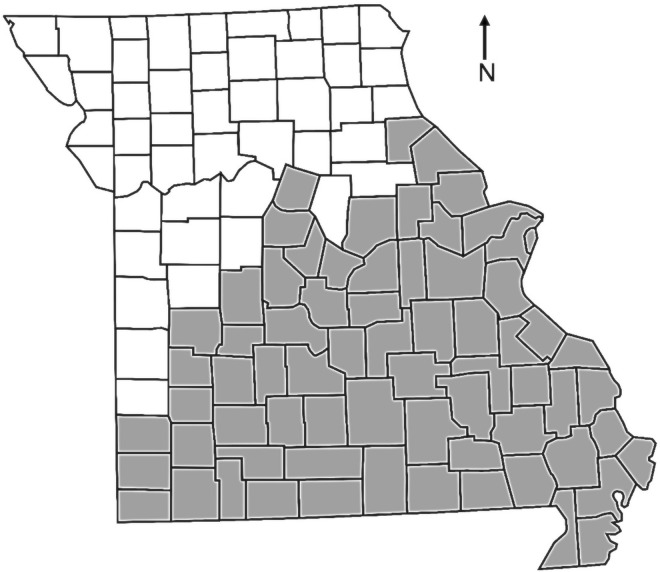
A county map of the ecoregions in Missouri: Great Plains (white) and Eastern Temperate Forest (grey).

As the objectives of this study were to assess the effects of environment on seasonality of the disease and the change in disease incidence over time, the data were divided into two categories based on Level I Ecoregions (Fig. 1) identified by the Missouri Herpetological Atlas Project (https://atlas.moherp.org), the ETF and the GP. Those counties that were composed of both ecoregions were placed into the ecoregion that comprised >50% of the county. Linear regression was used to determine if there was a significant difference in the rate of increase in confirmed cases of ehrlichiosis during this time between the two ecoregions.

To examine the seasonality of ehrlichiosis in each ecoregion, polynomial regression curve fitting was applied to each year of data. As such models are not reliable when the number of cases is too small to fit the curve, we arbitrarily selected to use years in which there were 20 or more cases in each ecoregion resulting in a total of 6 years (2007, 2008 and 2012–2015) and 857 total cases. To determine state-level seasonality for years with ⩾20 confirmed cases, all years after 2006 were included for a total of 9 years of data and 1090 cases. Disease confirmation data were provided by DHSS as numerical value from 1 to 52 (CDC Week) which is a standardised measurement to allow year-to-year comparisons of infectious disease data. For each year, a polynomial curve was fit to graphed data of confirmed cases over the 52-week period. Polynomial regression eliminates stochasticity and accounts for cases occurring earlier or later in the year than expected. After fitting the data with first-order through seventh-order polynomial functions, the sixth-order polynomial function was the best fit curve as determined by the *r*^2^-value. The peak week of the ehrlichiosis season was defined as the week corresponding to the curve maximum. The start and end weeks were defined as the weeks when the number of cases accelerates at the most rapid pace, and were found by finding the local maximum of the second derivative of the case curve before and after the determined peak week functions. Pairwise *t*-tests were conducted to determine if the average start, peak and end week of ehrlichiosis were significantly different between the two ecoregions.

## Results

**Fig. 2.**
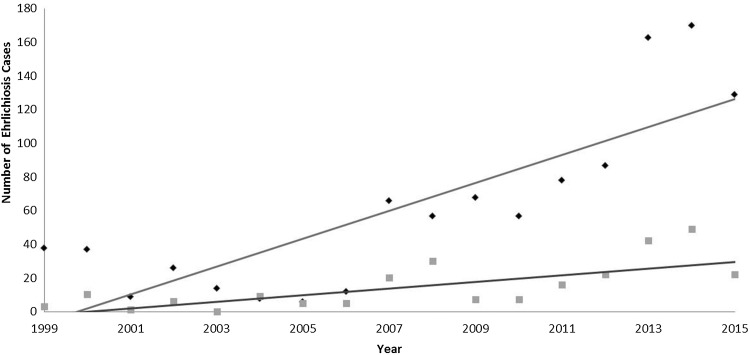
The number of confirmed ehrlichiosis cases from 1999 to 2015 in all of Missouri reported to by the Missouri Department of Health and Senior Services. Trend lines show the rate of change in two ecoregions: Great Plains (grey squares) and Eastern Temperate Forest (dark diamonds).

Since becoming notifiable in 1999, the number of confirmed ehrlichiosis cases has increased in both ecoregions in Missouri (Fig. 2). There were consistently more cases in the ETF ecoregion than in the GP ecoregion. There was a significant interaction between time and region (*P* = 0.000), suggesting that the incidence of ehrlichiosis cases has increased at a faster rate in ETF than in GP over the 15 years included in this study.

**Fig. 3.**
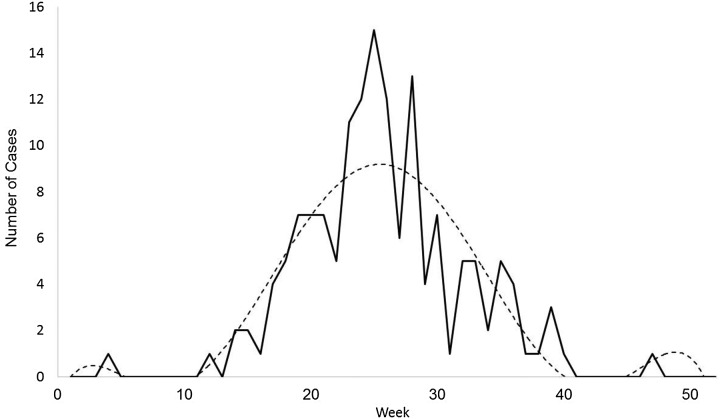
An example case curve for confirmed cases of ehrlichiosis in Missouri. This sample from 2015 shows the actual number of new case confirmations reported by the Missouri Department of Health and Senior Services each week throughout the year (solid line) and a sixth-degree polynomial function curve fitted to the data (dotted line).

The sixth-order polynomial captured the primary disease season and the outlying early and late cases (Fig. 3). The seasonal aspects of ehrlichiosis, as determined by the estimated start week, end week, peak week and duration, were not significantly different between the ecoregions (Table 1; all values of *P* > *α*/4 = 0.0125; Bonferroni correction). In Missouri, the estimated average ehrlichiosis season lasts 7.5 months beginning in the middle of March (week 11), peaking in the end of June (week 26) and ending in the beginning of October (week 41, Table 1).

**Table 1.** The seasonality and duration of ehrlichiosis for the Great Plains ecoregion and Eastern Temperate Forest ecoregion in Missouri

	Start week			Peak week			End week			Duration		
Year	GP^a^	ETF^b^	MO^c^	GP	ETF	MO	GP	ETF	MO	GP	ETF	MO
2007	12	12	12	28	27	28	44	42	43	32	30	31
2008	11	12	12	28	28	28	42	45	43	31	33	31
2009	–^d^	–	11	–	–	26	–	–	41	–	–	30
2010	–	–	11	–	–	26	–	–	41	–	–	30
2011	–	–	11	–	–	26	–	–	41	–	–	30
2012	9	11	10	25	26	25	41	41	41	32	30	31
2013	12	12	12	27	27	27	42	43	42	30	31	30
2014	11	11	11	27	27	27	42	42	42	31	31	31
2015	10	10	10	25	26	25	41	42	41	31	32	31
Mean (s.d.)	11.3 (0.8)	10.8 (1.2)	11.1 (0.8)	26.8 (0.8)	26.7 (1.4)	26.4 (1.1)	42.5 (1.4)	42.0 (1.1)	41.7 (0.9)	31.2 (1.2)	31.2 (0.8)	30.6 (0.5)

For each year, the seasonality is indicated by the CDC Week for the start, peak and end week of the season and duration is reported as the total number of weeks in the season.

a Great Plains ecoregion.

b Eastern Temperate Forest ecoregion.

c Missouri.

d Fewer than 20 cases in one and/or both ecoregion(s) for that year.

Variation in the season among years was due more to variation in the estimated end of the season as there was little variation among the estimated start of the season (Table 1). There was only 1 year, 2015, in which a case was diagnosed earlier than the estimated start of the season (Table 2). This case occurred 6 weeks prior to the estimated start week for that year and 7 weeks prior to the average estimated start week for the Missouri ehrlichiosis season. In contrast, all years except 2010 have cases diagnosed after the estimated end of the season (Table 2). In 2008 and 2013, the last cases of ehrlichiosis were diagnosed at the end of December (week 52), 11 weeks after the estimated end of the average Missouri ehrlichiosis season.

**Table 2.** The CDC Week for the first and last case of ehrlichiosis in Missouri based on the Missouri Department of Health and Senior Services reports for each year included in the seasonality study and the number of cases occurring outside the average estimated start and end week of each yearly season

Year	Week of first case	# of cases before start week	Week of last case	# of cases after end week
2007	16	0	44	1
2008	17	0	52	4
2009	13	0	43	1
2010	16	0	40	0
2011	17	0	47	2
2012	15	0	50	3
2013	18	0	52	3
2014	15	0	45	3
2015	4	1	47	1

## Discussion

Findings in this study suggest that the ehrlichiosis risk in Missouri is at least 7 months long with the disease season accelerating in mid-March peaking at the end of June and ending in mid-October. The average estimated start week of the disease season was before the first reported case in most years. As the symptoms of ehrlichiosis occur, a median of 9 days with a range of 5–14 days following infection by *E. chaffeensis* [1], our model alerts the public health workers that pathogen acquisition begins earlier then disease expression. Our models were only able to capture the end of the disease season in one of the 9 years in this study. This difference in the ability of curve fitting to capture the end of the season may be partly due to the lag between pathogen contraction and reporting. In addition to lag, it is also possible that the infections were contracted outside of the state of Missouri as reporting is based on county in which infection was diagnosed.

The estimated Missouri ehrlichiosis season overlaps with reported activity of the tick vector, *A. americanum.* In northeast Missouri, adult ticks have been reported to be active from March through July and nymphs from March to September [16]. In southeast Missouri, adult ticks have been reported to be active in all months except September, December and January and nymphs from March through November [7]. Behavioural diapause is absent in adult females [17] and likely in a portion of the nymphal population [18]. The absence of diapause enables ticks to become active when environmental conditions are favourable for questing suggesting the season may be extended in years with above average winter temperatures.

Our understanding of ehrlichiosis incidence is based on the number of confirmed cases that are voluntarily reported to the CDC. Although under-reporting has been suggested due to poor public and physician awareness of the disease [19, 20] and mild or asymptomatic infections [19], we observed an increase in the number of confirmed cases of ehrlichiosis in the state with a greater rate of increase in the ETF ecoregion since 1999. Increased awareness of the disease and diagnostic procedures has likely affected reporting. The observed spatial variation in the rate of increased ehrlichiosis cases may be skewed as each case report is by county of disease confirmation, not the county of pathogen acquisition.

Changes in the distribution and abundance of the tick vector *A. americanum* could also account for the observed increase in the number of confirmed cases of ehrlichiosis. The northern section of GP ecoregion was on the edge of the range of this species range, but our current understanding of the distribution of this species now includes Nebraska, Iowa and Michigan and this change is consistent with climate change [21]. Tick abundance may also be influenced by the habitat variation between the GP and ETF ecoregions. Consistent tick abundance data from across the state is not available as there is no state-wide vector surveillance programme. However, spatial variation of ehrlichiosis in Missouri is aggregated with most of the clusters of high incidence in the ETF region [22]. Social-ecological factors that were associated with incidence were low human population density and a high proportion of vacant housing and forest cover and increased density of white-tailed deer [22].

Knowing when tick-borne illnesses occur is important for the implementation of effective disease prevention methods. Awareness that the disease season is at least 7 months long, spanning multiple environmental seasons, and that there is potential for ticks to be actively host seeking on warm winter days is important for the prevention of tick-borne illness. A state-wide tick surveillance programme would provide information to better understand when and where tick-borne pathogens are more likely to be acquired.
